# ORP2 regulates free cholesterol accumulation in hepatocytes during MASH

**DOI:** 10.1097/HC9.0000000000000737

**Published:** 2025-07-14

**Authors:** Jin Wu, Yudi Zhao, Liwen Qiu, Qiaoli Chen, Xiaowei Wang, Jingwen Gu, Yan Liang, Yingjie Zhang, Hong-Yu Wang, Yang Liu, Xiaoqin Wu, Shuai Chen, Feng-Jung Chen, Mingming Gao, Hongyuan Yang

**Affiliations:** 1 Shanghai Key Laboratory of Metabolic Remodeling and Health, State Key Laboratory of Genetics ﻿and Development of Complex Phenotypes, Institute of Metabolism and Integrative Biology, School of Life Sciences, Department of Endocrinology and Metabolism, Zhongshan Hospital, Fudan University, Shanghai, China; 2 Department of Biochemistry and Molecular Biology, The Key Laboratory of Neural and Vascular Biology, Ministry of Education, The Key Laboratory of Vascular Biology of Hebei Province, Cardiovascular Medical Science Center, Hebei Medical University, Shijiazhuang, Hebei﻿, China; 3 MOE Key Laboratory of Model Animal for Disease Study, Department of Endocrinology, Nanjing Drum Tower Hospital, The Affiliated Hospital of Nanjing University Medical School, Model Animal Research Center, School of Medicine, Nanjing University, Nanjing, China; 4 Department of Integrative Biology and Pharmacology, University of Texas Health Science Center at Houston, Houston, Texas, USA; 5 University of Texas MD Anderson UTHealth Houston Graduate School of Biomedical Sciences, Houston, Texas﻿, USA; 6 School of Biotechnology and Biomolecular Sciences, The University of New South Wales, Sydney, New South Wales, Australia

**Keywords:** bile acids, free cholesterol accumulation, hepatic steatosis, MASH, ORP2

## Abstract

**Background:**

Cholesterol crystals in hepatocytes are known to strongly associate with human metabolic dysfunction–associated steatohepatitis. However, it remains unclear which molecular pathway(s) regulates free cholesterol accumulation and the formation of cholesterol crystals in hepatocytes. In cultured cell lines, oxysterol-binding protein-related protein 2 (ORP2) functions to deliver cholesterol to the plasma membrane from endosomal compartments.

**Methods:**

Here, we generated liver-specific *ORP2* knockout (ORP2-LKO) mice and characterized their metabolic phenotypes on chow and high-fat diet.

**Results:**

The ORP2-LKO mice developed much more severe hepatic steatosis than floxed control mice after high-fat diet feeding. They also demonstrated more severe liver inflammation and damage. Notably, free but not esterified cholesterol, as well as cholesterol crystals, accumulated in the ORP2-LKO liver. The expression of Cyp7a1 was significantly upregulated in the ORP2-LKO liver, accompanied by the accumulation of taurocholic acid. Our results thus unveil an important in vivo function of ORP2 in preventing free cholesterol from accumulating in the mouse liver.

**Conclusions:**

Our results suggest that impaired cholesterol trafficking may exacerbate the deposition of cholesterol crystals in hepatocytes, promoting the development of metabolic dysfunction–associated steatohepatitis.

## Graphical abstract

**Figure:**
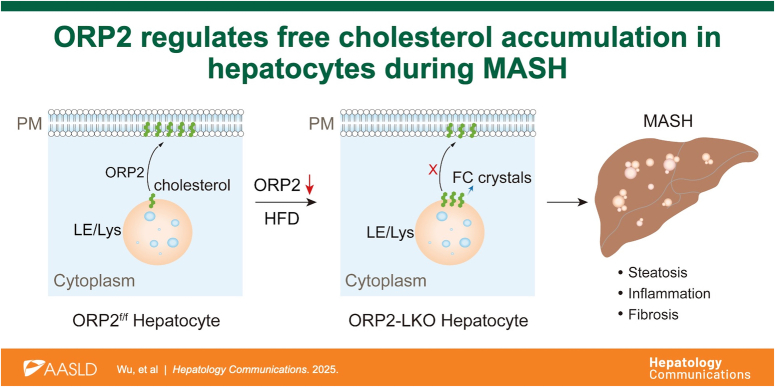

## INTRODUCTION

Cholesterol is an essential constituent of the organellar membranes of mammalian cells.^1^^–^^4^ Cholesterol is synthesized in the endoplasmic reticulum (ER); however, the concentration of cholesterol in the ER is very low (<5% of total ER lipids). Instead, up to 90% of cellular free cholesterol exists in the plasma membrane (PM), where it plays a critical role in maintaining the stability and functions of the PM.^5^ Endosomal compartments also have a relatively high concentration of cholesterol. Thus, the distribution and trafficking of cellular cholesterol are tightly controlled. The oxysterol-binding protein (OSBP) and OSBP-related proteins (ORP, for OSBP-related protein) have emerged as key mediators/regulators of lipid transport at contact sites between the ER and other organelles.^6^^–^^10^ Among the 12 OSBP/ORP family members, OSBP, ORP1, ORP2, and ORP4 transfer cholesterol in a manner dependent on phosphatidylinositol 4-phosphate and/or phosphatidylinositol 4, 5-bisphosphate.^8^^,^^11^ Previously, we demonstrated that ORP2 functions to deliver cholesterol to the PM from endosomal compartments in cultured cell lines.^11^ However, the roles of ORP2 in physiological and pathological contexts have not been extensively examined.

Accumulation of free cholesterol and cholesterol crystals in hepatocytes is strongly associated with human metabolic dysfunction–associated steatohepatitis (MASH).^12^^–^^14^ Hepatic free cholesterol concentration is determined by several well-known pathways, including endogenous synthesis, uptake of cholesterol-containing plasma lipoproteins, cholesterol excretion into bile, and conversion of cholesterol into bile acids.^15^ In addition, free cholesterol can be esterified into hydrophobic cholesterol esters for storage within cytoplasmic lipid droplets (LDs) and for secretion within ER luminal lipoproteins.^16^ Surprisingly, however, gene expression studies failed to detect significant changes in those well-established pathways regulating cholesterol homeostasis in hepatocytes from patients with MASH.^12^ Therefore, additional factors/pathways may control free cholesterol accumulation in hepatocytes during the development of MASH.

To examine the in vivo function of ORP2, we generated a liver-specific knockout mouse line of ORP2 (ORP2-LKO) in this study. We observed a significant accumulation of free cholesterol and cholesterol crystals in the liver of ORP2-LKO mice fed a high-fat diet (﻿HFD). Our results thus identify a novel pathway for controlling the distribution of free cholesterol during MASH development.

## METHODS

### Animal experiments

The *Osbpl2* ^f/f^ (ORP2^f/f^) mice were generated by Cyagen Biosciences, then bred with Alb-Cre transgenic mice to generate liver-specific knockout mice. The experimental group consisted of Alb-Cre^+^; ORP2^f/f^ (ORP2-LKO) mice, while littermate ORP2^f/f^ mice served as controls. These mice were obtained by mating Alb-Cre^+^; ORP2^f/f^ mice with ORP2^f/f^ mice. Mice were either maintained on a standard rodent chow diet for 9 months or placed on a high-fat fed for 8–10 weeks starting at 8 weeks of age. The HFD used in this study provided 60% of calories from fat, 20% from carbohydrates, and 20% from protein (D12492, Research DIETS, Xiao Shu You Tai (Beijing) Biotechnology). Mice were housed in the animal facility with 12-hour light/12-hour dark cycles, the temperature at 22 °C–23 °C and 20%–60% humidity with free access to diet and water. Male mice are used in the studies. All animal procedures were approved by the Laboratory Animal Ethical and Welfare Committee of Hebei Medical University, the Institutional Ethics Committee of Fudan University, and Nanjing University, under a permit for animal use in the Center of Experimental Animal at Shanghai and Nanjing. The permit followed the Experimental Animal Regulations set by the National Science and Technology Commission, China.

#### Body composition measurement

The minispec LF50 nuclear magnetic resonance analyzer (Bruker) was used to measure the body composition of mice according to the manufacturer’s instructions.

#### Glucose tolerance test and insulin tolerance test

For the glucose tolerance test, mice were fasted for 14–16 hours and then i.p. given glucose (2 g of glucose/kg). Blood glucose was measured at 0, 15, 30, 60, and 120 minutes after injection of glucose. For the insulin tolerance test, mice were fasted for 6 hours before experiments. Insulin (0.7 U/kg) was administered i.p., and blood glucose was measured at 0, 15, 30, and 60 minutes after injection.

#### Indirect calorimetry measurement

Metabolic cage analysis was performed in Comprehensive Laboratory Animal Monitoring System (CLAMS) (Columbus Instruments). Mice were acclimatized to the metabolic cages for 24 hours. Mice were then monitored for 2 days on food intake, body weight, oxygen consumption, carbon dioxide production, and locomotor activities. The respiratory exchange ratio (RER) is calculated as: RER = VCO_2_/VO_2_.

#### Detection of serological indicators

LabAssay Triglyceride (Wako, 290-63701), LabAssay Total cholesterol (Wako, 635-50981), free cholesterol assay kit (APPLYGEN, E1006), aspartate aminotransferase assay kit (AST) (Nanjing Jiancheng, C010-2-1), alanine aminotransferase assay kit (ALT, Nanjing Jiancheng, C009-2-1) were used to measure biochemical parameters﻿. RNA-sequencing (RNA-seq) and metabolomics of liver tissues were analyzed by Personalbio.

### RNA extraction and RT-qPCR analysis

Total RNA from the liver was extracted with Trizol reagent (15596026CN, ThermoFisher). Complementary DNA was synthesized using the reverse transcription kit (11151ES10, Yeasen). RT-qPCR was performed with Hieff qPCR SYBR Green Master Mix (11202ES03, Yeasen). The primers used for RT-qPCR are listed in Table 1.

**TABLE 1:** List of the primers used in this study

Primer	Sequence
*m36b4-F*	CACTGGTCTAGGACCCGAGAAG
*m36b4-R*	GGTGCCTCTGGAGATTTTCG
*mOsbpl2-F*	TTCTTTGATGCCGTTACAGGC
*mOsbpl2-R*	CTTCTGGTGAACATGGGAGC
*mCd68-F*	TGTCTGATCTTGCTAGGACCG
*mCd68-R*	GAGAGTAACGGCCTTTTTGTGA
*mTnfα-F*	GACGTGGAACTGGCAGAAGAG
*mTnfα-R*	TTGGTGGTTTGTGAGTGTGAG
*mIl1β-F*	GCAACTGTTCCTGAACTCAACT
*mIl1β-R*	ATCTTTTGGGGTCCGTCAACT
*mCxcl1-F*	CTGGGATTCACCTCAAGAACATC
*mCxcl1-R*	CAGGGTCAAGGCAAGCCTC
*mCol1a1-F*	TAAGGGTCCCCAATGGTGAGA
*mCol1a1-R*	GGGTCCCTCGACTCCTACAT
*mCol3a1-F*	CTGTAACATGGAAACTGGGGAAA
*mCol3a1-R*	CCATAGCTGAACTGAAAACCACC
*mCol4a1-F*	CTGGCACAAAAGGGACGAG
*mCol4a1-R*	ACGTGGCCGAGAATTTCACC
*mCol6a1-F*	AACAGGAATAGGAAATGTGACCC
*mCol6a1-R*	ACACCACGGATAGGTTAGGGG
*mTgfb1-F*	CTCCCGTGGCTTCTAGTGC
*mTgfb1-R*	GCCTTAGTTTGGACAGGATCTG
*mLoxl2-F*	ATTAACCCCAACTATGAAGTGCC
*mLoxl2-R*	CTGTCTCCTCACTGAAGGCTC
*mDesmin-F*	GTGGATGCAGCCACTCTAGC
*mDesmin-R*	TTAGCCGCGATGGTCTCATAC

### Western blot analysis

Approximately 100 mg of tissues were resuspended and homogenized with lysis solution (50 mM Tris, 150 mM NaCl, 1% NP-40, 0.5% sodium deoxycholate, and 5 mM EDTA containing protease inhibitors [P1005, Beyotime]) and lysed for 15 minutes on ice, and then supernatant was collected after centrifugation at 12,000 rpm for 15 minutes at 4 °C. Protein concentrations were quantified using the bicinchoninic acid protein assay kit (P0012, Beyotime). Equal amounts of proteins were loaded onto sodium dodecyl sulfate–polyacrylamide gel electrophoresis and transferred to polyvinylidene difluoride membranes. After blocking with 5% milk for 1 hour at room temperature, primary antibodies were diluted in primary antibody dilution buffer (P0023A, Beyotime) and incubated with membranes overnight at 4 °C. Membranes were washed 3 times with ﻿tris-buffered saline with tween 20 at room temperature for 6 minutes per wash. Secondary antibodies (horseradish peroxidase-conjugated goat anti–mouse IgG [115-035-003, Jackson] and horseradish peroxidase-conjugated goat anti–rabbit IgG [111-035-003, Jackson]) were diluted in 5% milk and incubated with membranes for 1 hour at room temperature. Western blot analysis was conducted using the Bio-Rad system. Quantification of band intensities was performed using ImageJ (NIH). The following primary antibodies were used for western blotting: rabbit polyclonal anti-OSBPL2 (14751-1-AP, Proteintech), mouse monoclonal-β-Tubulin (AC021, Abclonal), and mouse monoclonal GAPDH (AC002, Abclonal).

### Liver lipid extraction and assay

Approximately 100 mg of frozen liver tissue was homogenized in 1 mL of cold PBS using a homogenizer. Lipids were extracted with chloroform/methanol (2:1, v/v), then dried under nitrogen and re-dissolved in 0.5 mL of 3% Triton X-100.

Liver triglyceride (TG) content and total cholesterol (TC) content were measured using assay kits from Bio Sino, following the manufacturer’s instructions. Liver cholesteryl ester content was calculated as the difference between TC and free cholesterol levels. The assays for liver TC (E1015) and free cholesterol (E1027-105) content were performed using kits from Applygen, also according to the manufacturer’s instructions.

### Histological analysis

The liver and adipose tissues were fixed and embedded in paraffin. Tissue sections were cut at a thickness of 7 μm and stained with hematoxylin and eosin. In addition, after fixation, the liver was embedded in OCT (Sakura Finetek), and 7 μm-thick sections were obtained by cryo-sectioning. Lipid deposition in these cryo-sections was assessed using Oil-Red O staining. To detect free cholesterol, liver frozen sections were incubated with 50 μg/mL filipin (Sigma, F9765) for 30 minutes at room temperature and mounted without DAPI. Filipin fluorescent was visualized using confocal microscopy (Leica TCS SP5). Cholesterol crystals in frozen sections were observed under a polarized light microscope (SOPTO CX40P).

For paraffin-embedded sections, immunohistochemical analysis was performed using an F4/80 antibody (GB113373-100, Servicebio) to evaluate macrophage infiltration in the liver tissues. Sirius Red staining (Solarbio) was used to assess liver fibrosis.

Adipocyte quantification was conducted using ImageJ software. For each experimental group, 5 mice were analyzed, with 5 fields of view at ×200 magnification selected per mouse. The area of each adipocyte in these fields was measured and quantified.

### Flow cytometry analysis of liver Ly6C^high^ and Ly6C^low^ macrophages

Approximately 500 mg of liver tissue was disaggregated mechanically to isolate nonparenchymal cells. The cells were resuspended in 0.5% BSA solution and incubated at room temperature for 15 minutes to block nonspecific binding. The cells were stained with the following antibodies for 30 minutes: CD45-PerCP (14-0451-82), F4/80-PE (17-4801-82), CD11b-FITC (11-0112-82), and Ly6C-APC (17-5932-82) (all from Tonbo Biosciences). Then, the stained cells were analyzed using an Agilent flow cytometer. First, the CD45-positive cell population was gated﻿. Within the CD45-positive gate, the CD11b and F4/80 double-positive macrophages were further identified and selected. Then, the proportions of Ly6C^high^ and Ly6C^low^ cells were analyzed within the CD11b and F4/80 double-positive macrophage subset.

### Statistical analysis

Data were presented as mean ± SD. Statistical analyses were performed using GraphPad Prism 8 software. The significance of differences between the 2 groups was assessed using a 2-tailed Student *t* test. For multiple comparisons, 2-way ANOVA with a post-hoc Holm-Šidák test was utilized, with the *p* values multiplicity adjusted. Statistical difference was shown as *p < 0.05, **p < 0.01, and ***p < 0.001. ^NS^*p* > 0.05 represents no significant difference.

## RESULTS

### Generation and characterization of ORP2-LKO mice

**FIGURE 1:**
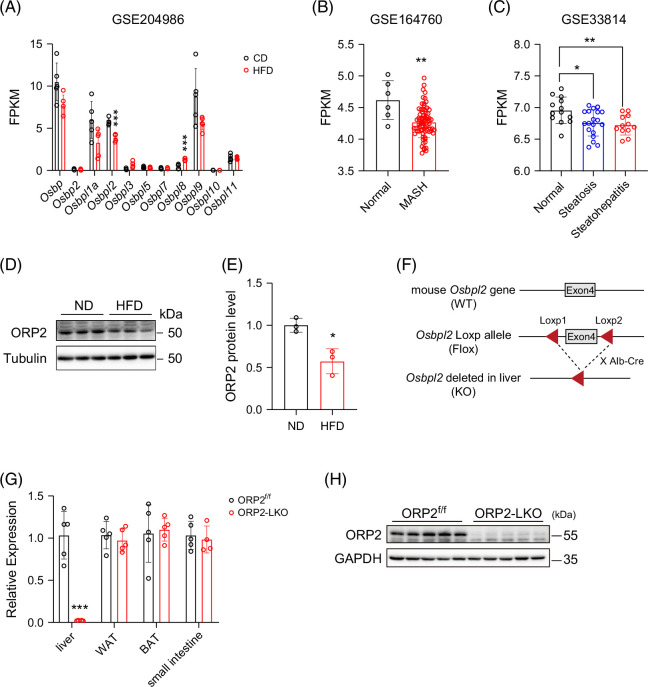
Association of ORP2 and fatty liver and construction of ORP2-LKO mice. (A) The FPKM mapped reads of ORP family members mRNA expression in the liver of mice treated with an HFD compared with those treated with an ND from the GEO database GSE204986 (*n* = 5). (B) The FPKM mapped reads of *OSBPL2* mRNA expression in the human liver tissue from healthy controls (normal, *n* *=* 6) and patients with MASH (*n* *=* 74) from the GEO database GSE164760. (C) The FPKM of *OSBPL2* mRNA expression in the human liver tissue surgical samples (normal, *n* *=* 13; steatosis, *n* *=* 19; steatohepatitis, *n* *=* 12) from the GEO database GSE33814. (D) Western blot analysis of ORP2 protein levels in the liver of ND mice and HFD mice. (E) Quantification of ORP2 protein level in (D) (*n* = 3). (F) Schematic diagram showing the construction of liver-specific *ORP2* knockout mice. (G) RT-qPCR analysis of relative mRNA expression of *ORP2* in tissues of ORP2^f/f^ and ORP2-LKO mice (*n* = 4–5). (H) Western blot analysis of ORP2 protein levels in the liver of ORP2^f/f^ and ORP2-LKO mice. Data are presented as mean ± SD. Statistical significance was determined using a 2-tailed Student *t* test. *p < 0.05, **p < 0.01, ***p < 0.001. Abbreviations: FPKM, Fragments Per Kilobase of transcript per Million; HFD, high-fat diet; MASH, metabolic dysfunction–associated steatohepatitis; ND, normal diet; ORP2, oxysterol-binding protein-related protein 2.

A database search revealed a significant decrease in the transcriptional level of *Osbpl2/ORP2* in mouse liver following HFD treatment compared with the normal chow diet (GSE204986) (Figure 1A).^17^ Similarly, analysis of 2 independent human liver GEO data sets (GSE164760 and GSE33814) revealed that *OSBPL2* mRNA expression was lower in steatosis and steatohepatitis liver tissues compared with normal controls (Figures 1B and C). Consistently, the protein level of ORP2 was significantly downregulated in the high-fat-diet–induced liver compared with the normal diet, as confirmed by western blot analysis (Figures 1D and E). To explore if and how *Osbpl2/ORP2* regulates the physiological function of the liver, we crossed *Osbpl2* ^f/f^ (ORP2^f/f^) mice with Alb-Cre mice and generated liver-specific *Osbpl2/ORP2* knockout mice, herein designated as ORP2-LKO mice (Figure 1F). The mRNA expression level of *Osbpl2* was dramatically and significantly decreased specifically in the liver (Figure 1G). As shown in Figure 1H, the protein level of ORP2 in the liver was almost completely depleted.

To determine the effect of liver ORP2 deficiency on whole-body physiology, we first measured the body and organ weights of 9-month-old mice fed a chow diet. No significant differences in body weight, organ weight, and fat weight were observed between ORP2-LKO and control (ORP2^f/f^) mice during the rearing period (Supplemental Figures S1A–C, http://links.lww.com/HC9/C37). In addition, plasma levels of TG, TC, ALT, and AST did not show notable differences between ORP2-LKO and ORP2^f/f^ mice (Supplemental Figures S1D–F, http://links.lww.com/HC9/C37). No obvious differences in lipid accumulation or inflammation in the liver were detected between ORP2-LKO mice and ORP2^f/f^ mice, as assessed by liver weight ratio (Supplemental Figure S1G, http://links.lww.com/HC9/C37), TG (Supplemental Figure S1H, http://links.lww.com/HC9/C37), and TC (Supplemental Figure S1I, http://links.lww.com/HC9/C37) content, as well as hematoxylin and eosin, Oil Red O, F4/80, and Sirius red staining analyses (Supplemental Figure S1J, http://links.lww.com/HC9/C37). These results suggest that the absence of liver ORP2 does not have a strong metabolic impact on whole-body and liver physiology under a normal chow diet.

### Absence of liver ORP2 exacerbates diet-induced obesity

**FIGURE 2:**
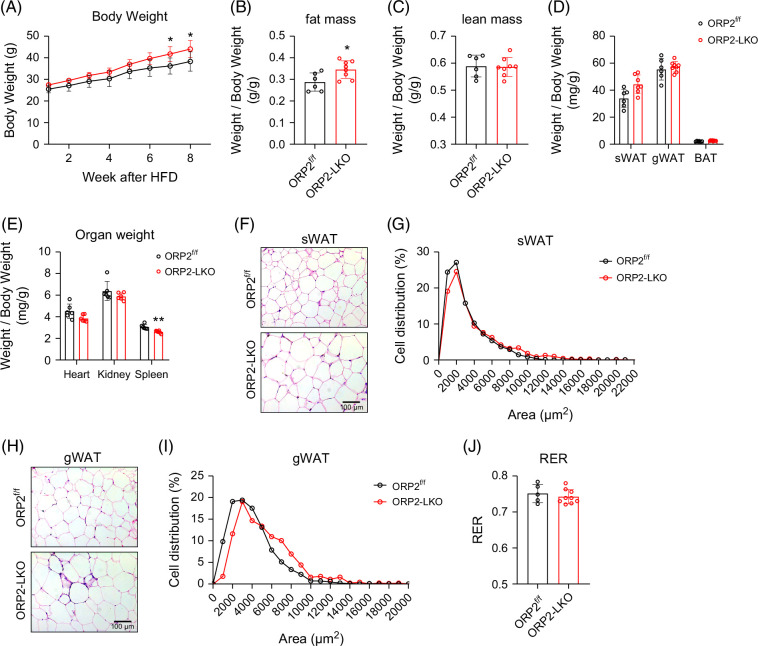
Absence of liver ORP2 exacerbated diet-induced obesity. (A) Growth curve of ORP2^f/f^ and ORP2-LKO mice during HFD feeding (*n* = 6–8). (B, C) Fat mass and lean mass of ORP2^f/f^ mice and ORP2-LKO mice measured by NMR analyzer after 8 weeks of HFD feeding (*n* = 6–8). (D) Fat weight of ORP2^f/f^ mice and ORP2-LKO mice (*n* = 6–8). (E) Organ weight of ORP2^f/f^ mice and ORP2-LKO mice (*n* = 5–6). (F, G) H&E staining images of subcutaneous fat (sWAT) and quantification of adipocyte size of sWAT in ORP2^f/f^ mice and ORP2-LKO mice. Scale bar, 100 µm. (H, I) H&E stain of gonadal fat (gWAT) and quantification of adipocyte size of gWAT in ORP2^f/f^ mice and ORP2-LKO mice. Scale bar, 100 μm. (J) RER of mice subjected to metabolic cage analysis (*n* = 5–9). Data are presented as mean ± SD. Statistical significance was determined using 2-way ANOVA, followed by Šidák’s multiple comparison test for (A) and 2-tailed Student *t* test for (B)–(E) and (J). *p < 0.05, **p < 0.01. Abbreviations: gWAT, gonadal white adipose tissue; H&E, hematoxylin and eosin; HFD, high-fat diet; NMR, nuclear magnetic resonance; ORP2, oxysterol-binding protein-related protein 2; RER, respiratory exchange ratio; sWAT, subcutaneous white adipose tissue.

To examine if liver ORP2 may play any role in HFD-induced metabolic abnormalities, 8-week-old ORP2-LKO and ORP2^f/f^ mice were fed HFD for 8 weeks. During the first few weeks of HFD treatment, weight gain in both ORP2-LKO mice and control mice (ORP2^f/f^) was comparable. However, after 7 weeks on HFD, ORP2-LKO mice gained significantly more weight than the control mice (Figure 2A). Body composition analysis by nuclear magnetic resonance showed that the fat mass of ORP2-LKO mice was significantly higher than that of control mice (Figure 2B), whereas the lean mass remained unchanged (Figure 2C). Among fat depots, the weight of subcutaneous white adipose tissue (sWAT) of ORP2-LKO mice increased slightly (Figure 2D), whereas the weight changes of the gonadal WAT (gWAT) and brown adipose tissue were not significant (Figure 2D). Spleen weight was moderately but significantly reduced in ORP2-LKO mice, with no significant differences in heart or kidney weights between ORP2^f/f^ and ORP2-LKO mice (Figure 2E). Interestingly, histological analysis showed that LDs were expanded in both sWAT (Figures 2F and G) and gWAT (Figures 2H and I) of ORP2-LKO mice fed with HFD. To further characterize these mice, we subjected them to metabolic cage analysis. The respiratory exchange ratio (Figure 2J), oxygen consumption (Supplemental Figure S2A, http://links.lww.com/HC9/C37), and carbon dioxide production (Supplemental Figure S2B, http://links.lww.com/HC9/C37) were similar between ORP2-LKO and ORP2^f/f^ mice. After 9–10 weeks on HFD, no significant differences were observed in glucose and insulin tolerance tests between ORP2-LKO and ORP2^f/f^ mice (Supplemental Figures S2C, D, http://links.lww.com/HC9/C37). Overall, these results indicate that the absence of liver ORP2 has a moderate impact on whole-body metabolism.

### Deficiency of liver ORP2 exacerbates HFD-induced liver steatosis

**FIGURE 3:**
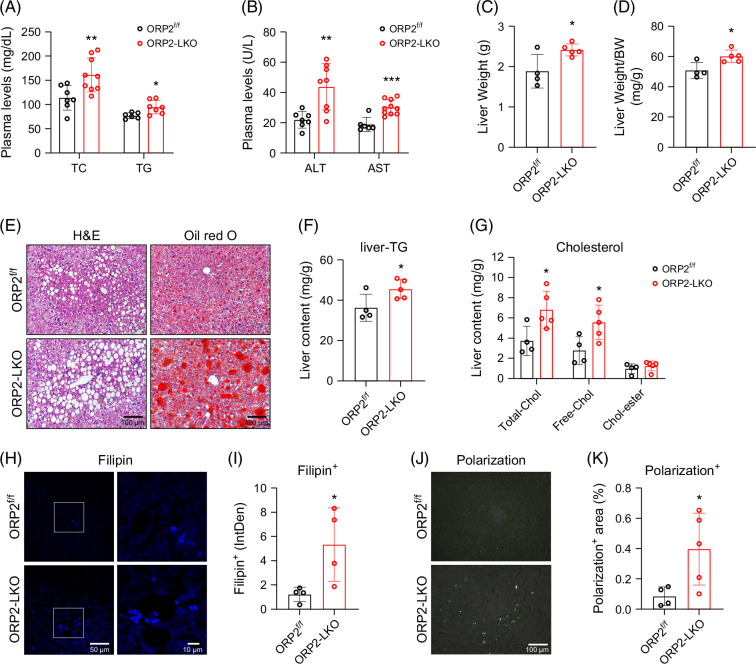
ORP2 deficiency exacerbated hepatic steatosis and free cholesterol accumulation. (A) Plasma levels of TC and TG in ORP2^f/f^ and ORP2-LKO mice after 8 weeks of HFD feeding (*n* = 6–10). (B) Plasma levels of ALT and AST in ORP2^f/f^ and ORP2-LKO mice after 8 weeks of HFD feeding (*n* = 6–10). (C, D) Liver weight (C) and the ratio of liver weight to body weight (D) of ORP2^f/f^ mice and ORP2-LKO mice (*n* = 4–5). (E) The H&E and Oil red O staining images of the liver of ORP2^f/f^ and ORP2-LKO mice. Scale bars, 100 μm. (F) The content of TG in the liver of ORP2^f/f^ and ORP2-LKO mice (*n* = 4–5). (G) The content of total cholesterol (Total-Chol), free cholesterol (Free-Chol), and cholesterol-ester (Chol-ester) in the liver of ORP2^f/f^ and ORP2-LKO mice (*n* = 4–5). (H, I) Representative Filipin staining images (H) and quantification of Filipin fluorescence intensity (I) in the liver section from ORP2^f/f^ and ORP2-LKO mice fed with HFD for 8 weeks (*n* = 4–5). Scale bars, 50 µm; (Enlarged) 10 µm. (J, K) Representative polarization microscopy images (J) and quantification of polarization positive area (K) in the liver sections from ORP2^f/f^ and ORP2-LKO mice fed with HFD for 8 weeks (*n* = 4–5). Scale bar, 100 μm. Data are presented as mean ± SD. Statistical significance was determined using a 2-tailed Student *t* test. *p < 0.05, **p < 0.01, ***p < 0.001. Abbreviations: H&E, hematoxylin and eosin; HFD, high-fat diet; ORP2, oxysterol-binding protein-related protein 2; TC, total cholesterol; TG, triglyceride.

The ORP2-LKO mice showed higher levels of plasma TC and TGs (Figure 3A), as well as higher levels of plasma ALT and AST, indicating the presence of liver injury (Figure 3B). Furthermore, the livers of ORP2-LKO mice were moderately but significantly heavier than those of control mice (ORP2^f/f^) (Figures 3C and D). As revealed by hematoxylin and eosin analysis and Oil red O staining, the ORP2-LKO liver developed severe hepatic steatosis, as characterized by vacuolated and lipid-laden hepatocytes (Figure 3E). Consistently, the TG (Figure 3F) and TC (Figure 3G) contents in the liver of ORP2-LKO mice were significantly elevated compared with control mice. Interestingly, the level of free cholesterol displayed a significant increase, whereas cholesterol-ester content remained similar between ORP2-LKO and control livers (Figure 3G). Filipin staining further confirmed the accumulation of free cholesterol in the livers of ORP2-LKO mice (Figures 3H and J). Consistent with the increased free cholesterol, more cholesterol crystals were observed in ORP2-LKO livers using polarized light microscopy (Figures 3J and K). Together, these results suggest that ORP2 functions to maintain normal lipid metabolism, especially cholesterol metabolism, in the liver during HFD-induced MASH development.

### Deletion of ORP2 exacerbates HFD-induced hepatic inflammation

**FIGURE 4:**
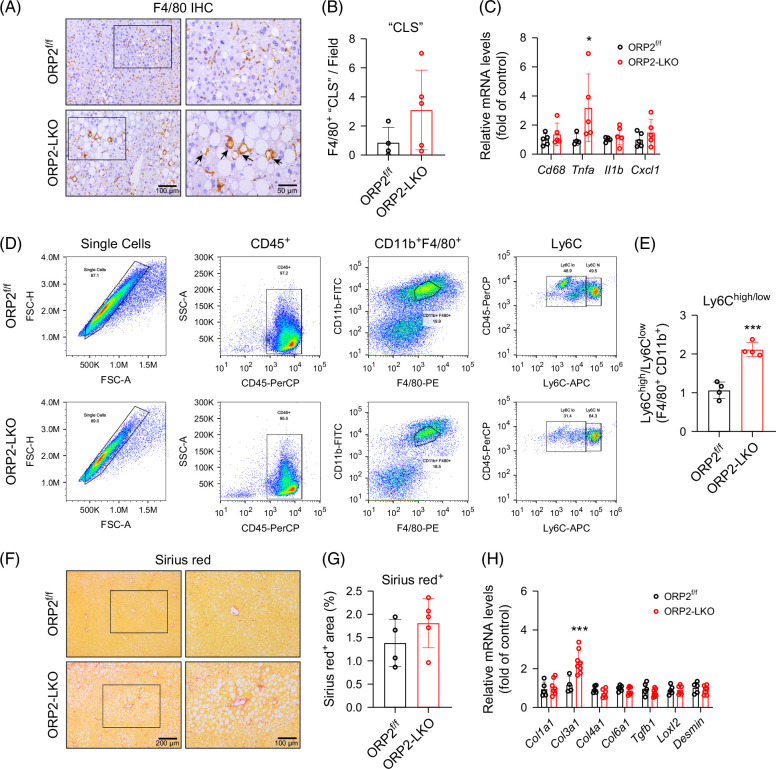
Severe hepatic inflammation in the liver of ORP2-LKO mice. (A, B) Representative immunohistochemical staining images of F4/80 (A) and quantification of F4/80 positive crown-like structure (B) in the liver section from ORP2^f/f^ and ORP2-LKO mice fed with a high-fat diet for 8 weeks (*n* = 4–5). Scale bars, 100 µm; (Enlarged) 50 µm. (C) RT-qPCR analysis of relative mRNA expression of inflammation genes in the liver of ORP2^f/f^ and ORP2-LKO mice fed with a high-fat diet for 8 weeks (*n* = 4–5). (D, E) Representative FACS analysis and quantification of Ly6C^high^/Ly6C^low^ macrophages gated on the F4/80^+^CD11b^+^ population within the CD45^+^ cells from the liver of ORP2^f/f^ and ORP2-LKO mice fed with a high-fat diet for 8 weeks (*n* = 4). (F, G) Representative Sirius red staining images (F) and quantification of Sirius red positive area (G) in liver sections from ORP2^f/f^ and ORP2-LKO mice fed with a high-fat diet for 8 weeks (*n* = 4–5). Scale bars, 200 µm; (Enlarged) 100 µm. (H) RT-qPCR analysis of relative mRNA expression of fibrosis genes in the liver from ORP2^f/f^ and ORP2-LKO mice fed with a high-fat diet for 8 weeks (*n* = 5–7). Data are presented as mean ± SD. Statistical significance was determined using a 2-tailed Student *t* test. *p < 0.05, ***p < 0.001. Abbreviation: ORP2, oxysterol-binding protein-related protein 2.

Many studies have reported that increased lipid or cholesterol accumulation in the liver can trigger inflammation and fibrosis.^13^^,^^18^ To investigate the role of ORP2 in liver inflammation during HFD treatment, we conducted a series of experiments in ORP2^f/f^ and ORP2-LKO mice. First, F4/80 staining revealed significantly stronger staining in the liver of ORP2-LKO mice compared with controls, with an increased number of crown-like structures (Figures 4A and B). In addition, the expression levels of inflammation-related genes, such as *Tnfα,* were significantly higher in the liver following *ORP2* deletion (Figure 4C). Flow cytometry analysis further revealed a significant increase in the population of Ly6C^high^ proinflammatory macrophages in the liver of ORP2-LKO mice (Figures 4D and E). However, despite these inflammatory changes, Sirius Red staining showed no significant difference in collagen deposition between ORP2-LKO and control mice (Figures 4F and G), although the mRNA levels of *Col3a1* were notably increased in ORP2-LKO livers (Figure 4H). Together, these findings suggest that the deletion of liver ORP2 exacerbates HFD-induced liver inflammation, although its impact on fibrosis appears limited.

### ORP2 is essential for normal bile acid metabolism in the liver

**FIGURE 5:**
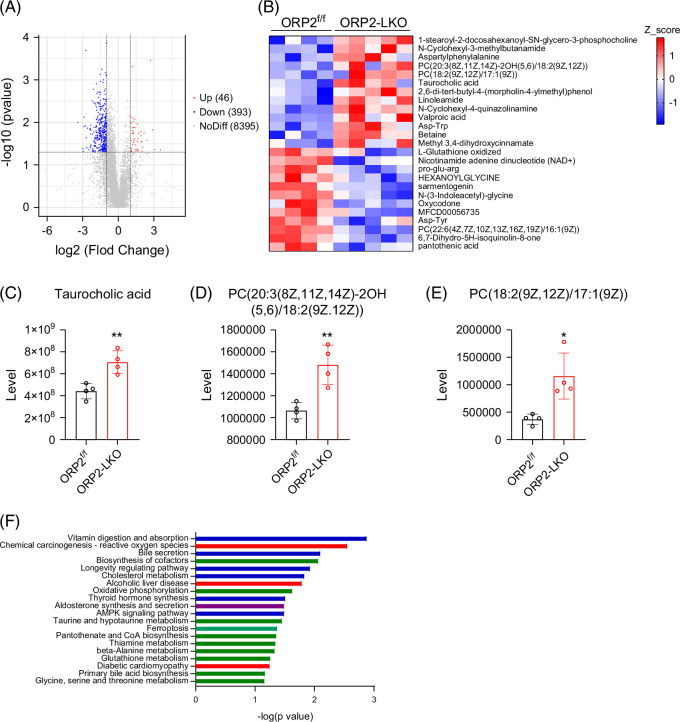
Metabolomics analysis of livers from ORP2^f/f^ and ORP2-LKO mice fed with a high-fat diet. (A) Volcano map of metabolomics analysis of the liver collected from ORP2^f/f^ and ORP2-LKO mice. Forty-six metabolites were upregulated, 393 metabolites were downregulated, and 8395 metabolites had no difference between ORP2-LKO and control mice (*n* = 4–5). (B) Heatmap of metabolomics of the liver in ORP2^f/f^ and ORP2-LKO mice (*n* = 4–5). (C–E) The level of taurocholic acid, PC (20:3/18:2), and PC (18:2/17:1) in the liver of ORP2^f/f^ and ORP2-LKO mice (*n* = 4). (F) KEGG pathway enrichment analysis of metabolomics results in the liver of ORP2^f/f^ and ORP2-LKO mice. Data are presented as mean ± SD. Statistical significance was determined using a 2-tailed Student *t* test. *p < 0.05, **p < 0.01. Abbreviations: KEGG, Kyoto Encyclopedia of Genes and Genomes; ORP2, oxysterol-binding protein-related protein 2; PC, phosphatidylcholine.

To gain additional insights into how ORP2 may control hepatic metabolism, we performed metabolomics and RNA-seq analyses on liver tissues from ORP2^f/f^ and ORP2-LKO mice fed with HFD. The metabolomics analysis revealed that 46 metabolites were increased, while 393 metabolites were decreased in the liver of ORP2-LKO mice compared with controls (Figure 5A). Notably, bile acid metabolites showed significant differences between the 2 groups (Figure 5B). Specifically, taurocholic acid, a key bile acid component, was significantly increased in ORP2-deficient liver (Figure 5C). In the bile, bile acids, phosphatidylcholine (PC), and cholesterol form mixed micelles, which are stored in the gallbladder.^19^ Metabolomics analysis also revealed that the levels of 2 species of PC, PC (20:3(8Z,11Z,14Z)-2OH(5,6)/18:2(9Z,12Z)) and PC (18:2(9Z,12Z)/17:1(9Z)), were increased markedly in the liver of ORP2-LKO mice compared with that of the control mice (Figures 5D and E). Kyoto Encyclopedia of Genes and Genomes (KEGG) pathway enrichment analysis of the metabolomics data revealed strong enrichment of genes involved in bile secretion and cholesterol metabolism (Figure 5F). These results suggest that ORP2 may regulate bile acid metabolism through its role in maintaining cellular cholesterol homeostasis.

**FIGURE 6:**
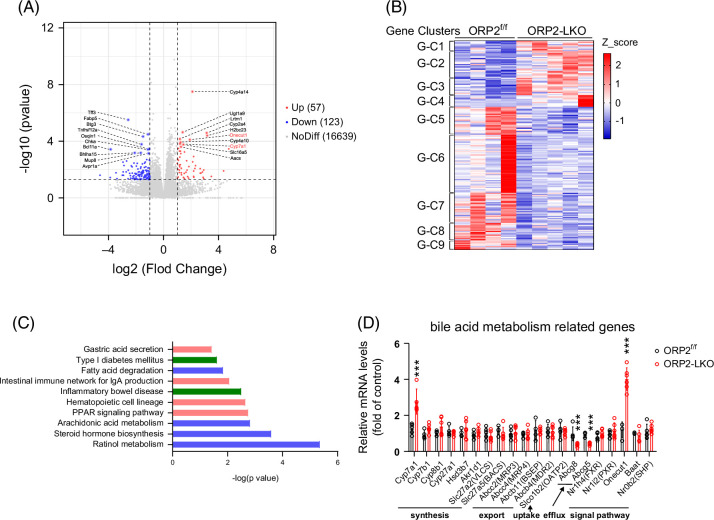
RNA-sequencing analysis of livers from ORP2^f/f^ and ORP2-LKO mice fed with a high-fat diet. (A) Volcano map of RNA-sequencing results of the liver collected from ORP2^f/f^ and ORP2-LKO mice. Fifty-seven genes were upregulated, 123 genes were downregulated, and 16,639 genes had no difference between ORP2-LKO and control mice (*n* = 4–5). (B) Heatmap of RNA-sequencing analysis of the liver in ORP2^f/f^ and ORP2-LKO mice. Gene cluster1 (G-C1): Histidine metabolism, G-C2: Retinol metabolism (fatty acid degradation), G-C3: Primary bile acid biosynthesis, G-C4: Arachidonic acid metabolism, G-C5: Linoleic acid metabolism, G-C6: Cytokine-cytokine receptor interaction (inflammatory bowel disease), G-C7: Cytokine-cytokine receptor interaction (hypertrophic cardiomyopathy), G-C8: Maturity onset diabetes of the young, and G-C9: Linolenic acid metabolism (*n* = 4–5). (C) KEGG pathway enrichment analysis of RNA-sequencing results in the liver of ORP2^f/f^ and ORP2-LKO mice. (D) Relative mRNA levels of bile acid metabolism–related genes analyzed by RT-qPCR (*n* = 4–6). Data are presented as mean ± SD. Statistical significance was determined using a 2-tailed Student *t* test. ***p < 0.001. Abbreviations: KEGG, Kyoto Encyclopedia of Genes and Genomes; ORP2, oxysterol-binding protein-related protein 2.

To further evaluate the effect of ORP2 on liver metabolism, we performed RNA-seq on liver mRNA from ORP2-LKO and control mice. Among the 16,819 genes analyzed, 57 genes were upregulated in ORP2-LKO mice, including the bile acid–related genes, such as *Cyp7a1* and *Onecut1* (Figure 6A). The RNA-seq heatmap showed significant upregulation of genes involved in primary bile acid biosynthesis (Gene Cluster 3, G-C3) (Figure 6B). KEGG pathway enrichment analysis of the RNA-seq data revealed a strong enrichment of genes involved in steroid biosynthesis, such as steroid hormone biosynthesis (Figure 6C). To confirm these results, we detected the expression of some key genes responsible for bile acid synthesis, export, uptake, efflux, and signaling pathway through qPCR. We found that the expression of *Cyp7a1* (bile acid synthesis) and *Onecut1* (bile acid signaling) was dramatically increased. By contrast, the expression levels of *Abcg5* and *Abcg8,* which are critical for cholesterol efflux, were significantly decreased in the ORP2-deficient liver (Figure 6D). In addition, although not statistically significant, *Cyp8b1* expression showed a clear upward trend, which may suggest increased production of CA and/or TCA (Figure 6D). Taken together, ORP2 deficiency led to disturbances in bile acid metabolism and increased bile acid accumulation in the liver.

## DISCUSSION

Increased free cholesterol content in the liver is one of the hallmarks of human MASH.^20^ Moreover, free cholesterol crystals were specifically found in patients with MASH, but not in patients with simple steatosis.^12^ Thus, crystals of free cholesterol may causally contribute to the development of MASH. For instance, free cholesterol may promote MASH through the stabilization and activation of TAZ.^21^ Although the important role of free cholesterol in the development of MASH has been well recognized, much is to be learned about the genetic factors and molecular mechanisms that govern the hepatic accumulation of free cholesterol during MASH development.

Previously, we and others demonstrated that the primary function of ORP2 is to deliver free cholesterol from endosomal compartments to the PM.^11^^,^^22^^,^^23^ ORP2 deficiency leads to the accumulation of free cholesterol in the endolysosomal membranes of cultured cell lines. In the current study, we show that upon HFD treatment, ORP2 deficiency caused significant free cholesterol accumulation in the liver, accompanied by increased inflammation and liver damage. It is conceivable that ORP2 deficiency in hepatocytes may cause free cholesterol to also accumulate in the endolysosomal membranes. The accumulated free cholesterol may then move directly to LDs for storage, as suggested.^12^ Upon HFD feeding, more and more cholesterol enters the endolysosomal system of hepatocytes, accumulates on the LD surface, and ultimately exceeds the capacity of LD surface phospholipids to accommodate cholesterol. Consequently, free cholesterol may then precipitate and form crystals next to LDs. Since less cholesterol can reach the PM of ORP2-deficient hepatocytes, cholesterol efflux may be reduced, as suggested by the decrease in ABCG5/8 expression, further exacerbating intracellular cholesterol accumulation. The increase in CYP7A1 expression is likely a response to increased cellular free cholesterol. Finally, it is important to note that the HFD used in this study is not a typical MASH-inducing diet and generally does not cause hepatic accumulation of free cholesterol, as it is not supplemented with cholesterol.^21^ Future work with MASH-inducing diets may lead to more profound pathological changes in the ORP2-deficient liver. Nevertheless, our data here strongly﻿ suggest that the loss of *ORP2* in hepatocytes significantly accelerates free cholesterol accumulation and crystallization.

There are a number of limitations in this study. For instance, we cannot determine if endolysosomal cholesterol is increased in ORP2-deficient hepatocytes. It is also somewhat confusing why cholesterol esters did not accumulate in ORP2-deficient hepatocytes. Perhaps the excessive free cholesterol is not efficiently esterified, or the cholesterol esters are more efficiently incorporated into lipoproteins and secreted in ORP2-deficient hepatocytes. The increased plasma TC supports the latter possibility. Moreover, we cannot explain why only taurocholic acid, but not other bile acids, is selectively upregulated in the ORP2-deficient liver. Future studies will address the underlying molecular mechanisms of the observations reported here.

In summary, we have generated a mouse model to examine ORP2 function in vivo, and our results are consistent with the known biochemical functions of ORP2. Most importantly, we identify ORP2 as a novel molecule that regulates the accumulation of hepatic free cholesterol during the development of MASH. Our results, therefore, indicate that proper intracellular cholesterol trafficking may represent an important regulatory node to prevent free cholesterol accumulation in hepatocytes during MASH.

## Supplementary Material
