# Supplementary material for: ORP2 regulates free cholesterol accumulation in hepatocytes during MASH
Source: Hepatol Commun. 2025 Jul 14;9(8):e0737. doi: 10.1097/HC9.0000000000000737 (PMC12262990; doi:10.1097/HC9.0000000000000737)

Supplemental Figure 1. Metabolic characterization of ORP2 LKO mice fed with chow diet.

A. Growth curve of ORP2$^{f/f}$ and ORP2-LKO mice fed with normal diet (*n* = 6-8).

B. Organ weight of ORP2$^{f/f}$ and ORP2-LKO mice fed with normal diet (*n* = 6-8).

C. Fat weight of ORP2$^{f/f}$ and ORP2-LKO mice fed with normal diet (*n* = 6-8).

D and E.

Plasma level of total cholesterol (TC) and triglyceride (TG) in ORP2$^{f/f}$ and ORP2-LKO mice at 3, 6, and 9 months of age (*n* = 6-8).

F. Plasma level of ALT and AST in ORP2$^{f/f}$ and ORP2-LKO mice at 9 months of age (*n* = 6-8).

G. The ratio of liver weight to body weight of ORP2$^{f/f}$ and ORP2-LKO mice (*n* = 6-8).

H and I.

The content of TG and TC in the liver of ORP2$^{f/f}$ and ORP2-LKO mice (*n* = 6-8).

J. The H&E staining, Oil red O staining, F4/80 immunofluorescence staining, and Sirius red staining images of the liver in ORP2$^{f/f}$ and ORP2-LKO mice. Scale bars, 100 μm.

Data are presented as mean ± SD. Statistical significance was determined using two-way ANOVA was performed, followed by Šidák's multiple comparison test for (A) and a two-tailed Student's *t*-test for (B)-(I).

Figure S1

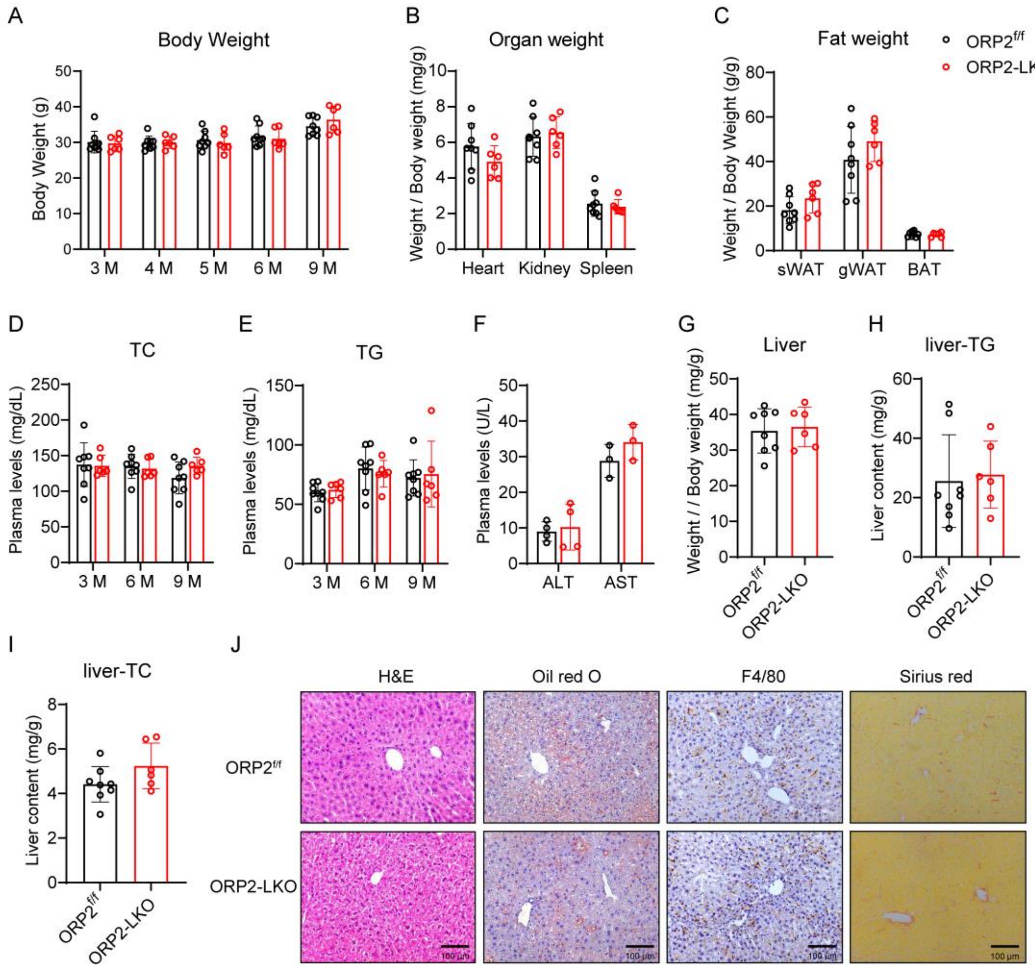

Supplemental Figure 2. Systemic metabolic homeostasis, glucose tolerance, and insulin sensitivity of ORP2-LKO mice fed with high-fat diet.

A and B.

VO$_2$ (A) and VCO$_2$ (B) of ORP2$^{f/f}$ and ORP2-LKO mice subjected to metabolic cage analysis fed with a high-fat diet for 9 weeks. (*n* = 5-9).

C. Glucose tolerance test (GTT) of ORP2$^{f/f}$ and ORP2-LKO mice fed with a high-fat diet for 9 weeks. (*n* = 6-9).

D. Insulin tolerance test (ITT) of ORP2$^{f/f}$ and ORP2-LKO mice fed with a high-fat diet for 10 weeks. (*n* = 6-9).

Data are presented as mean ± SD. Statistical significance was determined using two-way ANOVA was performed, followed by Šidák's multiple comparison test.

Figure S2

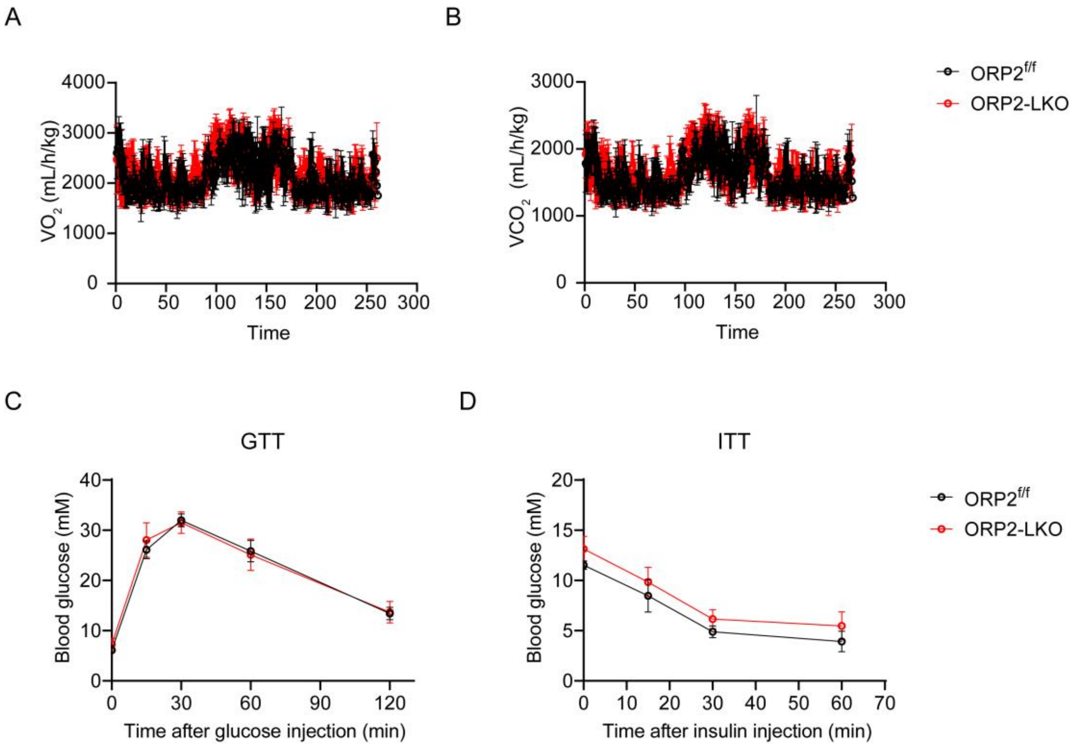

Supplement: Supplementary file 1 [file hc9-9-e0737-s001.pdf]
